# Experimental and theoretical study of the effect of different functionalities of graphene oxide/polymer composites on selective CO_2_ capture

**DOI:** 10.1038/s41598-022-20189-5

**Published:** 2022-09-26

**Authors:** Branislav Stankovic, Iranzu Barbarin, Oihane Sanz, Radmila Tomovska, Fernando Ruipérez

**Affiliations:** 1 POLYMAT and Departamento de Química Aplicada, Facultad de Ciencias Químicas, University of the Basque Country UPV/EHU, Joxe Mari Korta Zentroa, Tolosa Hiribidea, 72, 20018 Donostia-San Sebastián, Spain; 2 Faculty of Physical Chemistry, University of Belgrade, Studentski Trg 12-16, Belgrade, 11050 Republic of Serbia; 3 IKERBASQUE, Basque Foundation for Science, María Díaz de Haro 3, 48013 Bilbao, Spain; 4 POLYMAT and Physical Chemistry Department, Faculty of Pharmacy, University of the Basque Country, 01006 Vitoria-Gasteiz, Spain

**Keywords:** Theory and computation, Organic-inorganic nanostructures, Environmental sciences, Chemistry, Chemical engineering, Materials chemistry, Physical chemistry, Theoretical chemistry

## Abstract

There is a constant need for versatile technologies to reduce the continuously increasing concentration of CO_2_ in the atmosphere, able to provide effective solutions under different conditions (temperature, pressure) and composition of the flue gas. In this work, a combination of graphene oxide (GO) and functionalized waterborne polymer particles was investigated, as versatile and promising candidates for CO_2_ capture application, with the aim to develop an easily scalable, inexpensive, and environmentally friendly CO_2_ capture technology. There are huge possibilities of different functional monomers that can be selected to functionalize the polymer particles and to provide CO_2_-philicity to the composite nanostructures. Density functional theory (DFT) was employed to gain a deeper understanding of the interactions of these complex composite materials with CO_2_ and N_2_ molecules, and to build a basis for efficient screening for functional monomers. Estimation of the binding energy between CO_2_ and a set of GO/polymer composites, comprising copolymers of methyl methacrylate, n-butyl acrylate, and different functional monomers, shows that it depends strongly on the polymer functionalities. In some cases, there is a lack of cooperative effect of GO. It is explained by a remarkably strong GO-polymer binding, which induced less effective CO_2_-polymer interactions. When compared with experimental results, in the cases when the nanocomposite structures presented similar textural properties, the same trends for selective CO_2_ capture over N_2_ were attained. Besides novel functional materials for CO_2_ capture and a deeper understanding of the interactions between CO_2_ molecules with various materials, this study additionally demonstrates that DFT calculations can be a shorter route toward the efficient selection of the best functionalization of the composite materials for selective CO_2_ capture.

## Introduction

Significant and continuous augmentation of the concentration of greenhouse gases in the atmosphere has turned into one of the most fundamental and persistent problems nowadays because fossil fuel reserves are still affordable and developing countries are in the process of economic growth. Among various greenhouse gases, carbon dioxide (CO_2_) is a key player in the global-warming scenario^1^. Even though the global CO_2_ capture capacity has reached 40 million tons by 2020, gigatonnes per year of CO_2_ have to be captured to have a significant impact on climate change^2^. Since green energy technologies are far away from the point to replace fossil fuel energy sources, the reduction of CO_2_ emissions and, thus, the reduction of global warming, is one of the most challenging environmental issues nowadays. Therefore, the development of efficient, selective, and low-cost carbon-capture technologies is crucial^2^. Strategies such as chemical/physical adsorption^3^, enzymatic conversion^4^, and membrane separation^5^ have emerged as potential solutions.

Various adsorbents have been proposed for CO_2_ capture^6–8^, namely, porous polymers, ion-exchange resins, covalent- and metal-organic frameworks, zeolites, silica- and alumina-based materials, metal oxides, etc. However, most of them suffer from low adsorption capacities (or a long time is required for saturation), lack of good chemical/thermal stability and/or selectivity relative to other gases, or they have reduced activity in the presence of moisture, such as zeolite-based adsorbents^9, 10^.

Carbon-based adsorbents are arising as a promising alternative to overcome most of the mentioned drawbacks, owing to one of the highest adsorption capacities and relatively low energy requirements for regeneration^6–8^. Furthermore, features such as large surface area, stability in cycle operations, porous structure that can be easily functionalized, and fast adsorption kinetics endorse them as one of the most promising adsorbents. Among these materials, due to lower production costs, graphene and its derivatives have been considered for commercial use^11^. Aimed to further improve the adsorption capacity and separation ability, functionalization of graphene surface with heteroatoms (N, S, O, etc.) has been widely investigated, as well as the production of composites with polymers such as polypyrrole^12^, polyaniline^13^, polyindole^14^, polythiophene^15^, mono-, di-, and triethylene-triamine^16^, tetraethylenepentamine^17^, poly(diallyldimethylammonium chloride)/polystyrene sulfonate^18^, poly(dimethylsiloxane)^19^, polyether block amide^20^, polyethylene-imine^21^, and also with metal–organic frameworks^22^.

The addition of polymer nanoparticles onto graphene platelets, together with the functionalization of the surface, improves the physicochemical properties and provides easier handling. Such composite platelets are more durable and present improved stability in cycle operations. The polymer particles are usually produced by emulsion polymerization of different combinations of (meth)acrylic monomers, functionalized onto the surface by using a minor amount of functional monomers during the synthesis. The selection of highly CO_2_-philic functional monomers may be decisive towards the development of efficient composite platelets for CO_2_ selective adsorption and, thus, the screening and evaluation of different monomers is a fundamental step^23, 24^.

Computational chemistry has been revealed as a powerful tool in materials science^25, 26^. In particular, density functional theory (DFT) calculations may provide a detailed insight into the interactions between CO_2_ and different molecules, giving useful information for the pursuit of potential new adsorbents. The first computational studies, regarding the interaction of CO_2_ and graphene, were performed even before the successful isolation of graphene. Cinke et al.^27^ used benzene and coronene as a model of graphene to estimate its interaction with CO_2_. Allouche and Ferro^28^ performed mixed quantum mechanics/molecular mechanics ONIOM (B3LYP:UFF) calculations on a moderate-sized cluster model of graphene. According to their results, CO_2_ can not be adsorbed. Xu et al.^29^ did more precise ONIOM calculations (B3LYP:DFTB-D) on a smaller cluster. They obtained small interaction only after correction on zero-point vibrational energies. Cabrera-Sanfelix^30^ found that CO_2_ can be better physisorbed on top of vacancies in the graphene structure. Liu and Wilcox^31^ performed more detailed calculations and stated that the CO_2_ binding energy on a monovacancy is 4-fold higher than that on the defectless graphene. The same authors also showed that hydroxyl and carbonyl groups in graphene establish stronger interactions with CO_2_ due to higher electron densities^32^. Sun et al.^33^ showed improved adsorption near nitrogen atoms in N-doped graphene and, by means of energy decomposition analyses, demonstrated the relevance of dispersion interactions in this process. Wang et al.^34^ performed a similar study on N and O co-doped graphene and established that the heteroatoms bind CO_2_ more strongly. They also highlighted the relevant role of dispersion interactions. Dasgupta et al.^35^ used higher levels of theory and small molecular models to estimate the binding energy between graphene functionalized with different groups and CO_2_. Seema et al.^15^ synthesized S-doped microporous carbon materials by chemical activation of reduced graphene oxide/polythiophene (rGO/PTh) and showed, using DFT calculations, that the interaction energy of CO_2_ with thiophene is higher than that with pyrrole, in agreement with the observed higher adsorption capacity of rGO/PTh. These authors suggested that the reason for this stronger attraction is due to a larger negative charge of S in thiophene than that of N in pyrrole. Finally, polymeric systems have also been considered. Patel et al.^36^ calculated the binding energies for CO_2_ adsorption on several azo-bridged covalent organic polymers. Although simple models were used, accurate trends in binding energy were obtained. In summary, quantum chemical calculations confirm the well-known fact that carbon materials doped with heteroatoms show enhanced CO_2_ adsorption. Das et al.^37^ investigated the role of surface OH groups in triazine-based N-rich porous organic polymers for enhancing CO_2_ capture. Using the ωB97XD density functional method, capable of a reliable description of both short-range and long-range interactions, with, a relatively small 3-21G* basis set, they found that CO_2_ mainly interacts through the N–N⋯O and O–H⋯O hydrogen bonds, but also through the weak N⋯C, N–O⋯C, and C–H⋯O interactions. Ullah et al.^38^ performed a combined experimental and theoretical study of CO_2_ adsorption by amine and amide porous polymers. Using the B3LYP-D3/6-311++G** level of theory they obtained a moderate agreement with experiments due to the different morphologies of samples.

As has been previously mentioned, GO/polymer composites are very promising candidates for commercial applications, and the selection of optimal functional monomer can significantly increase adsorption capacity. However, comprehensive investigation, both experimental and theoretical, of the effects that different functionalities of GO/polymer composites have on selective CO_2_ capture are sparse. Even more, to the best of our knowledge, there is no data in the literature on the theoretical estimation of binding energy for the CO_2_ adsorption on these composites. Thus, in this work, we have performed quantum chemical calculations to estimate the binding energy of CO_2_ with a set of GO/polymer composites. More precisely, in the pursuit of new materials for CO_2_ capture, we investigated copolymers of methyl methacrylate (MMA), *n*-butyl acrylate (BA), and different functional monomers. Furthermore, the functional monomers, which, according to the theoretical study provided the strongest interactions with CO_2_, have been used for the synthesis of functionalized MMA/BA particles and combined with graphene oxide platelets. The synthesis of composites is specially designed to give a reliable platform for comparison with the theoretical results. Finally, the synthesized composites have been evaluated experimentally for CO_2_ capture. Besides, a similar study has been performed for N_2_ to evaluate the CO_2_/N_2_ selectivity of these materials. The observed experimental trends of CO_2_ adsorption capacities are in accordance with the trends of computed binding energies, demonstrating that the DFT calculations are a useful tool for the development of new materials for CO_2_ capture. Additionally, it is convenient to say that our procedure for synthesis has advantages over many similar reports since it is done by low energy synthesis procedure (using ascorbic acid as redox initiators under mild conditions, i.e. without any solvents, high temperature, and C-footprint treatments, nor the use of aggressive acid–base treatments). Having that in mind, it is aligned with the green chemistry trends and has the prospect to scale up well.

## Computational details

All geometry optimizations were performed within density functional theory (DFT) by using the long-range corrected ωB97XD functional^39^ together with the 6–31+G(d) basis set. For the calculations including the GO model, the smaller 6–31G(d) basis set was used to relieve the computational effort. The zero-point vibrational energies (ZPVE) were evaluated within the harmonic oscillator approximation at the same level of theory. All optimized structures showed no imaginary frequencies, meaning that all structures are minima on the potential energy surface. The CO_2_ and N_2_ molecules were placed in different positions near every functional group of adsorbents and the structure with the lowest energy (highest binding energy) is presented. Also, when the structure of composites was optimized, the polymer was placed so that as many interactions between functional groups of GO and polymer are achieved. No less than six rearrangements were checked. The electronic energies were then refined by single-point calculations performed on the optimized structures using the 6–311++G(2df,2p) basis set. All calculations were carried out with the Gaussian 16 package^40^.

As a compromise between computational cost and accuracy, the model of GO is constructed to be the one that can guarantee that the polymer remains within the line which connects centroids of outer benzene rings. Graphene is randomly functionalized with two carboxyls, two epoxies, and one hydroxyl group in order to approximate the average functionalization of the real GO, that is, to achieve an adequate ratio between carbon and other atoms, as well as the uniformity of the distribution. Graphene edges were fully terminated by hydrogen atoms. Lengths of the same types of bonds in the interior of the graphene plane and near the edges were similar, indicating that edge effects are not significantly pronounced. In particular, the molecular formula for the model of the GO platelet was C_69_H_22_O_7_ (Supplementary Fig. S1). This is the smallest molecular model that would allow including the main interactions between the GO platelet and both the functional monomer and CO_2_/N_2_.

The pursuit of the global energy minimum for the GO/monomer/CO_2_ and GO/monomer/N_2_ clusters would demand the exploration of a huge conformational space, which is out of our present computational capabilities. Thus, in order to find the most stable configuration, only several conformations were considered, where the CO_2_ and N_2_ molecules were located in different positions, near every functional group. Besides, the functional monomer was arranged in such a way that as many interactions as possible were established with the GO platelet. The energy differences between all the conformations explored were very small, and the structure with the lowest energy is used for the discussions.

## Experimental part

### Materials

An aqueous dispersion of graphene oxide sheets of 4 mg mL^−1^ (Graphenea) was used as supplied. The monolayer content in the dispersion was > 95% and in a pH range between 2.2 and 2.5. The elemental analysis of graphene oxide layers was provided in the technical data sheet from Graphenea: C (49–56%), H (0–1%), N (0–1%), S (2–4%), and O (41–50%). Technical monomers, methyl methacrylate (MMA, Quimidroga) and butyl acrylate (BA, Quimidroga), were used as supplied without any further purification. Sodium 4-vinylbenzenesulfonate (NaSS, Sigma-Aldrich), glycidyl methacrylate (GMA, Acros Organics), 2-hydroxyethyl methacrylate (HEMA, Sigma-Aldrich), and 2-aminoethyl methacrylate hydrochloride (AEMH, Sigma-Aldrich) were used as functional monomers. Tert-butyl hydroperoxide solution (TBHP, Sigma-Aldrich) and l-ascorbic acid (AsA, Sigma-Aldrich) were employed as redox initiators. Furthermore, sodium dodecyl sulfate (SDS, Sigma-Aldrich) and hexadecyltrimethyl ammonium chloride (HAC, Sigma-Aldrich) were employed as emulsifiers. Sodium bicarbonate (NaHCO_3_, Sigma-Aldrich) was used as a buffer. Deionized water was used throughout the experimental work.

### Synthesis of polymer nanoparticles

The batch emulsion polymerization process was used for the synthesis of functionalized polymer nanoparticles in aqueous dispersion (polymer latex). As main monomers, MMA and BA in 50/50 weight ratio were used, to which 3 wt% of functional monomer was added, i.e. NaSS, GMA, HEMA, and AEMH. In all cases, the same formulation was employed as described in Table 1, with a solids content of the final aqueous dispersions of 20 wt%. Two different types of surfactants were used, SDS in GMA, HEMA, and NaSS systems and HAC in AEMH system.

**Table 1:** Formulation for the synthesis of latexes.

Compounds	Amount (g)
MMA	16.49
BA	16.49
FM	1.02
Surfactant	1.02
NaHCO_3_	0.85
TBHP	0.34
AsA	0.34
Water	136

The reactions were performed in a 250 mL jacketed glass reactor equipped with a reflux condenser, temperature probe, nitrogen and feeding inlet, and stainless-steel stirrer rotating at 200 rpm. Pre-emulsion (monomers, surfactant, buffer, and water) was charged in the reactor, and then the temperature was raised to 70 °C, after which an aqueous solution of TBHP initiator was added as a shot, whereas AsA was fed for 120 min into the reactor. The reaction mixture was then kept at 70 °C for an additional 30 min before cooling to room temperature.

### Synthesis of composite GO/polymer platelets

Composite platelets were prepared by simple blending of aqueous dispersions of GO and polymer particles (1:0.5 weight ratio of GO:polymer), which were left agitated overnight at room temperature. Afterward, the hybrid dispersion was subjected to a freeze-drying process, in Telstar LyoQuest 55 at − 49 °C and 0.2 mbar for 3 days. To check how much polymer was incorporated onto the GO platelets, the aqueous phase of dispersion was analyzed gravimetrically. The agitation of GO and polymer mixture was stopped at different time periods. When the agitation was stopped, the platelets precipitated and the residual aqueous phase was analyzed. 2 mL of the dispersion extracted at different times was dried in the oven. The polymer quantity present in the aqueous phase was calculated from the difference in weight of wet samples. It was found that after 3 h agitation of the dispersion mixture of GO and polymer, there was no polymer left in the dispersion, or in other words, all added polymer was completely incorporated onto the GO platelets.

This method of synthesis was selected in order to prevent the development of complex, hierarchical porous morphology, typical for these systems, as we reported previously^23, 24^, expecting to produce composite platelets that differ just in the functionalization, introduced by the selected functional monomers. In this way, a solid platform for comparison with the theoretical models used for the calculations was provided.

### Characterization

The z-average particle size (*d*_z_) of the polymer particles was measured by Dynamic Light Scattering Spectroscopy (DLS), using the Malvern Zetasizer Nano ZS. Before the measurement, a fraction of the latex was sufficiently diluted with deionized water in order to avoid multiple scattering. Analyses were carried out at a temperature of 25 °C. The reported particle sizes are the average of three repeated measurements per sample.

The gel contents of the polymer particles (fraction of polymer insoluble in THF due to the presence of crosslinked and branched polymer chains) were determined by Soxhlet extraction. A few drops of latex were placed on glass fiber square pads and dried overnight at 60 °C. Then, the filter, together with the dried polymer, was subjected to a continuous extraction with THF under reflux in the Soxhlet for 24 h. After that, the filter was dried overnight at 60 °C in order to weigh the gel polymer fraction.

The molecular weights corresponding to the soluble fraction of the polymers were determined by gel permeation chromatography (GPC). The soluble part from the Soxhlet extraction was first dried, redissolved in GPC grade THF at a concentration of 2 mg mL^−1^, and finally, the solution was filtered (polyamide Ф = 45 μm) before injection into the GPC instrument via an autosampler (Waters 717). The GPC consisted of a pump (LC-20A, Shimadzu), a differential refractometer (Waters 2410), and three columns in series (Styragel HR2, HR4, and HR6, with pores sizes ranging from 10^2^ to 10^6^ Å). The chromatograms were obtained at 35 °C using a THF flow rate of 1 mL min^−1^. The equipment was calibrated using narrow polystyrene standards, thus, the presented molecular weights are relative to this standard.

In terms of graphene-polymer composite materials, thermal stability and the amount of oxygen-containing functional groups presented in the monolithic structure were studied by the TGA500 apparatus (TA instrument). Samples of around 2 mg were heated under a nitrogen atmosphere (90 mL min^−1^) from 25 to 800 °C, at a rate of 10 °C min^−1^.

The surface morphology of the composites was analyzed by scanning electron microscopy (SEM) using Hitachi TM3030 scanning electron microscope at 15 kV after the samples were coated with a thin gold layer, whereas the structure of the composites was analyzed by transmission electron microscopy (TEM) using Tecnai TM G2 20 Twin device at 200 kV (FEI Electron Microscopes). Prior to analysis, the samples were embedded in epoxy resin, from which ultra-thin sections (80 nm) were cut with a diamond knife on Leica EMFC6 ultramicrotome device and placed on a 200 mesh copper grid.

The textural properties of the monoliths were characterized by N_2_ adsorption–desorption, performed at − 196 °C in a Micromeritics ASAP 2020. Prior to the measurements, the samples were degassed at 110 °C for 8 h under vacuum. From N_2_ adsorption–desorption isotherms, the specific surface area and the adsorption average pore width (4 V/A) were calculated from the Brunauer–Emmett–Teller (BET) equation. Moreover, the t-plot method was used to estimate the micropore volume. Finally, the pore volume was calculated using the method proposed by Barrett–Joyner–Halenda. The CO_2_ adsorption capacities of the 2D graphene-polymer composites were determined using a TGA analyzer. Prior to the adsorption measurements, the samples were heated to 100 °C in an N_2_ atmosphere at a flow of 50 mL min^−1^ and held at that temperature for 30 min. The samples were then allowed to cool to 25 °C. Once the temperature reached 25 °C, the gas was changed to pure CO_2_ at a flow rate of 50 mL·min^−1^ until a constant weight was observed. The weight change of the sample was interpreted as the CO_2_ adsorption capacity. The same measurements were performed for N_2_ adsorption. These values were corrected by taking into account the buoyancy effect of gas change during the measurements.

## Results and discussion

**Figure 1:**
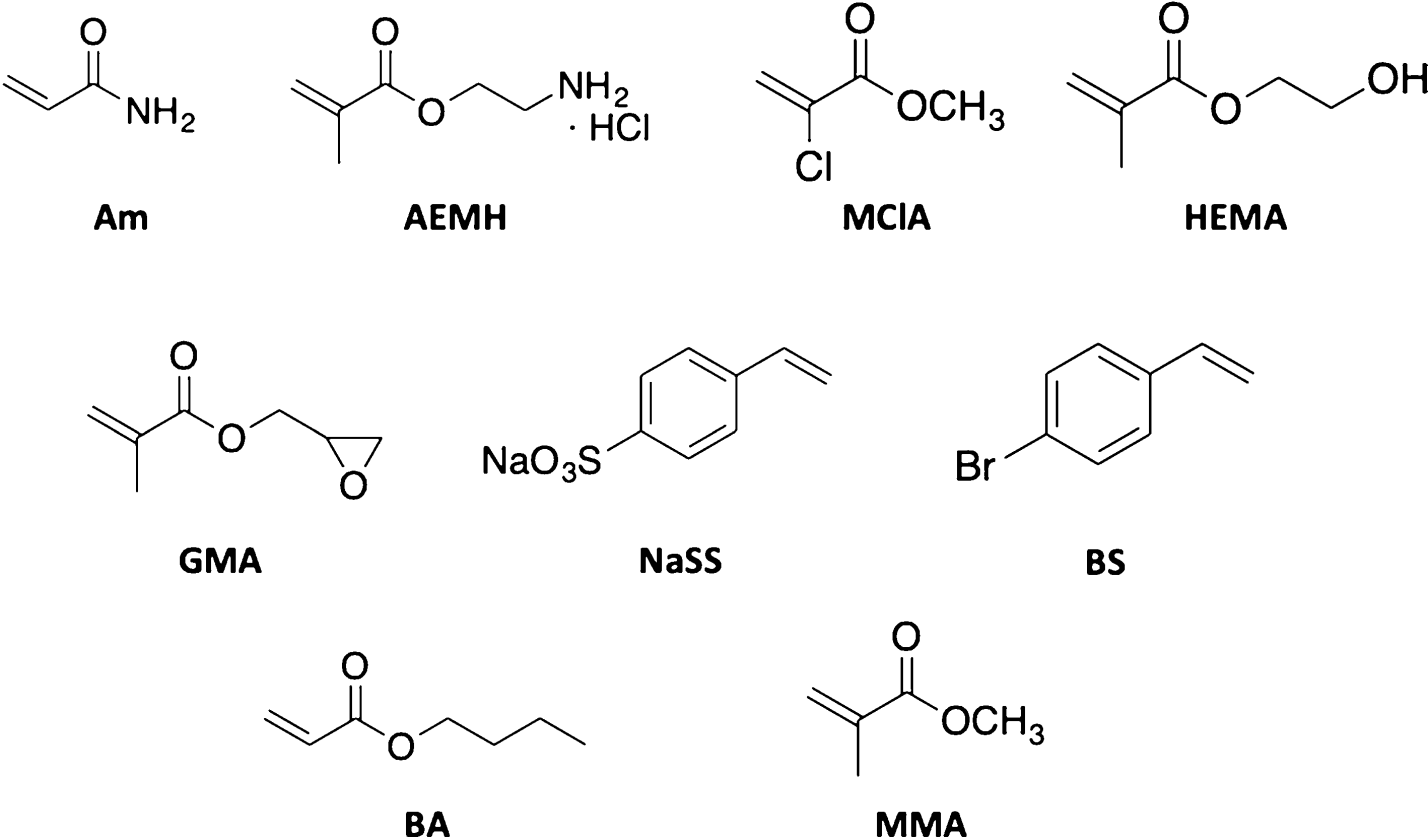
Molecular structures of the monomers: acrylamide (Am), 2-aminoethyl methacrylate hydrochloride (AEMH), methyl 2-chloro acrylate (MClA), hydroxyethyl methacrylate (HEMA), glycidyl methacrylate (GMA), sodium 4-vinylbenzenesulfonate (NaSS), 4-bromostyrene (BS), butyl acrylate (BA), and methyl methacrylate (MMA).

In this work, a set of functional monomers, namely, acrylamide (Am), 2-aminoethyl methacrylate hydrochloride (AEMH), 4-bromostyrene (BS), 2-hydroxyethyl methacrylate (HEMA), glycidyl methacrylate (GMA), methyl 2-chloro acrylate (MClA), and sodium 4-vinylbenzenesulfonate (NaSS), see Fig. 1, have been computationally studied in the pursuit of new materials to improve the CO_2_ adsorption capacity of GO/polymer composites. To this end, the binding energy of CO_2_ in these materials has been estimated using the theoretical methods of quantum chemistry and the most promising composites have been evaluated experimentally.

The results are organized as follows: (1) the computational studies comprise the analysis of the binding of CO_2_ with the bare functional monomers, then with MMA/BA/monomer copolymers, and finally with GO/copolymer composites. These composites have also been used to analyze the binding energy for N_2_. (2) The experimentally evaluated CO_2_ and N_2_ capture abilities of selected composites.

### Computational analysis

The theoretical investigation was divided into four parts. In the first part, we calculated the binding energies between a set of functional monomers and CO_2_. In the second one, it was examined how the introduction of MMA and BA monomers affects the values of binding energy and the trends between them. In the third part, for some of the functional monomers, we estimated the binding energy between CO_2_ and GO/MMA/BA/monomer composites, as well as the interaction between the GO platelet and the copolymer. Finally, in the last part, the previously selected composites were used to study the binding energy with N_2_.

#### Interaction between CO_2_ and functional monomers

**Figure 2:**
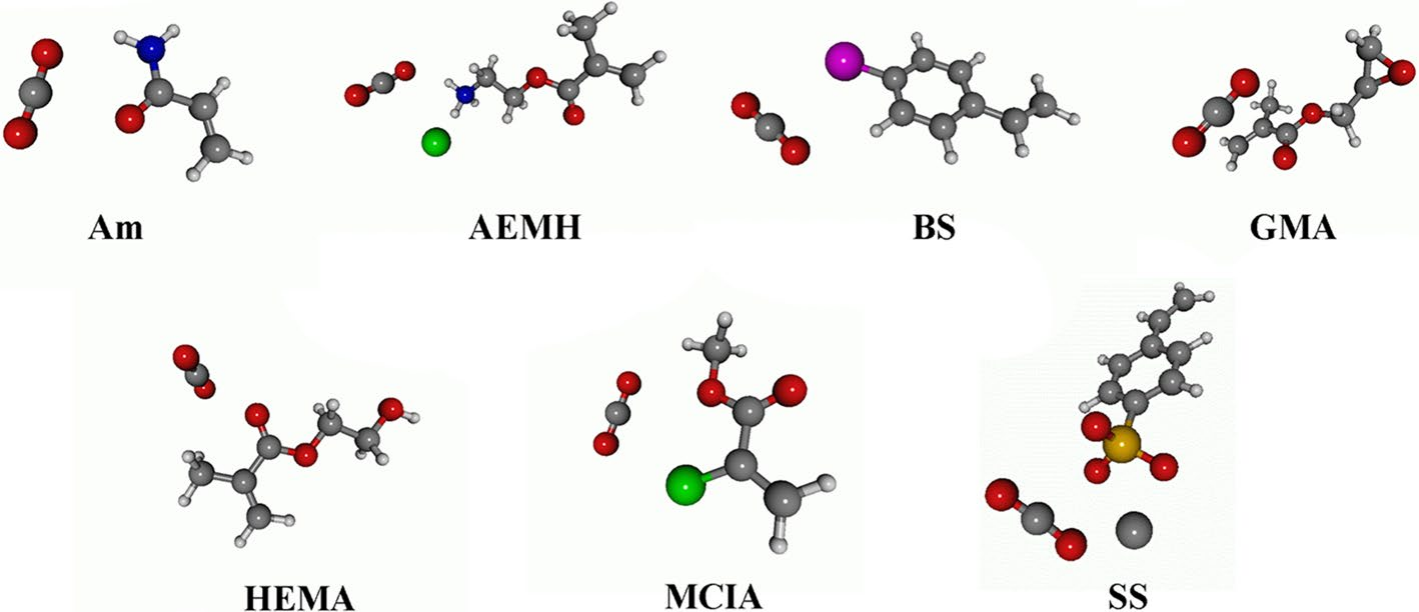
Optimized geometries of the CO_2_-monomer complexes.

In this subsection, the CO_2_ affinity of the functional monomers represented in Fig. 1 was analyzed in terms of binding energies and geometrical features. In Table 2 are collected binding energies and selected geometrical parameters of the complexes formed by CO_2_ and the functional monomers. The optimized geometries are represented in Fig. 2. The binding energy was estimated, using the ZPVE-corrected energies (*E*_*0*_), as:

1 $$\Delta E_{{{\text{CO}}_{2} }} = E_{0} ({\text{complex}}) - E_{0} ({\text{monomer}}) - E_{0} ({\text{CO}}_{2} )$$

**Table 2:** Binding energy of CO_2_ with functional monomers (Δ*E*_CO2_), in kJ·mol^−1^. Distance between CO_2_ and the monomers (*D*), in Å. C=O bond length (*R*_i_), in Å, and O=C=O angle (*α*), in degrees, of CO_2_ molecule.

	Am	AEMH	BS	GMA	HEMA	MClA	NaSS
Δ*E*_CO2_	− 16.1	− 7.2	− 5.5	− 10.7	− 12.7	− 5.5	− 29.1
*D*	2.241	2.934	3.328	3.111	3.037	3.292	3.072
*R*_1_	1.169	1.172	1.164	1.163	1.166	1.166	1.174
*R*_2_	1.161	1.158	1.166	1.166	1.164	1.164	1.156
*α*	176.3	176.0	179.0	178.5	177.5	179.0	176.2

These energies include the correction of the basis set superposition error (BSSE) by means of the counterpoise method^41, 42^.

Inspecting Table 2, it is observed that the weakest interactions with CO_2_ correspond to BS and MClA monomers (− 5.5 kJ·mol^−1^, for both of them). As a consequence, the C=O bond lengths of CO_2_ remain almost unchanged compared to the free molecule (1.165 Å) and the O=C=O angle deviates slightly from linearity (179.0° in both cases). Also, the CO_2_-monomer distances (*D*) are longer than for other monomers.

The highest binding energy corresponds to NaSS (− 29.1 kJ·mol^−1^), which is reflected in a larger deformation of CO_2_ molecule (*R*_1_ = 1.174 Å, *R*_2_ = 1.156 Å and *α* = 176.2°). Also, the distance between S=O groups and CO_2_ is relatively small considering the presence of the sodium cation. Furthermore, the relative position of this cation with respect to the S=O groups is modified after the introduction of CO_2_.

The remaining four functional monomers show intermediate binding energies. Again, for larger binding energies, larger deformations of CO_2_ and, in general, smaller distances between this molecule and the monomer are observed. The only clear exception is AEMH, which shows one of the lowest binding energies (− 7.2 kJ·mol^−1^), but a rather small CO_2_-monomer distance (*D* = 2.934 Å) and remarkable changes in CO_2_ geometry (*R*_1_ = 1.172 Å, *R*_2_ = 1.158 Å and *α* = 176.0°). This monomer comprises a molecular complex between the HCl and the NH_2_ moiety. After the adsorption of CO_2_, the molecular geometry of AEMH is significantly changed; the NH_2_-HCl interaction is remarkably affected, in such a way that the acidic proton is almost transferred to the amino group (the N–H distance is shortened from 1.612 to 1.460 Å, while the H–Cl distance is enlarged from 1.377 to 1.717 Å). Thus, the capacity of the nitrogen atom to interact with CO_2_ is decreased, which is reflected in the low binding energy. Finally, AEMH and NaSS are the only functional monomers for which geometry is changed with the adsorption of CO_2_, due to the presence of weakly bonded molecules or ions.

#### Interaction between CO_2_ and copolymers containing MMA/BA

In this subsection, the CO_2_ affinity of copolymers composed of methyl methacrylate (MMA), butyl acrylate (BA), and the functional monomers are evaluated. The introduction of MMA/BA in the system notably increases the binding energy for CO_2_ in all cases (between 4 and 12 kJ·mol^−1^, depending on the functional monomer), except for Am, see Table 3. This may be explained by the presence of the carbonyl groups of the MMA/BA copolymer, which allows additional interactions with CO_2_. The molecular structures of the copolymer-CO_2_ complexes are in the Supplementary Information (Supplementary Figs. S2–S8). It is convenient to say that values of calculated energies are in the range of those which can be found in the literature for CO_2_ adsorption by polymers^7, 36, 37^, which indicates that the size of the model and level of theory are adequate.

**Table 3:** Binding energy of CO_2_ with MMA/BA/monomer copolymers (Δ*E*_CO2_), in kJ·mol^−1^. Distance between CO_2_ and the polymer (*D*), in Å. C=O bond length (*R*_i_), in Å, and O=C=O angle (*α*), in degrees, of CO_2_ molecule.

	Am	AEMH	BS	GMA	HEMA	MClA	NaSS
Δ*E*_CO2_	− 13.4	− 14.7	− 9.5	− 18.3	− 24.2	− 14.7	− 33.4
*D*	3.008	2.230	3.697	3.038	2.208	3.074	3.072
*R*_1_	1.163	1.164	1.165	1.164	1.167	1.167	1.174
*R*_2_	1.167	1.150	1.165	1.166	1.163	1.163	1.156
*α*	176.7	176.5	179.2	177.0	177.4	178.2	175.8

The decrease observed in Am-containing copolymer with respect to the free Am (from − 16.1 to − 13.4 kJ·mol^−1^) can be ascribed to the interaction of the NH_2_ of Am with the C=O of BA (see Supplementary Fig. S2). Now, the amino group is less available to interact with CO_2_ and, consequently, CO_2_ is displaced closer to the carbonyl group of Am. For AEMH, the presence of MMA/BA allows CO_2_ to locate in a position closer to the amino group, and several bonds in AEMH change significantly (see Supplementary Fig. S3). For BS, the introduction of MMA and BA also changes the positioning of CO_2_, which is now located in a parallel plane over the benzene ring of BS, allowing for π–π interactions that increase the binding energy (see Supplementary Fig. S4). In the case of MClA, CO_2_ is now located relatively far from the Cl atom and closer to the carbonyl group, enhancing notably the affinity (see Supplementary Fig. S5). For the GMA system, CO_2_ is located between the C=O group of BA and the epoxide group of GMA (see Supplementary Fig. S6). The CO_2_-BA distance is smaller than the CO_2_-GMA one in the free monomer. As a consequence, the binding energy is increased. Similar to the previously discussed cases, in copolymer with HEMA, CO_2_ is adsorbed both by BA and HEMA. In the free monomer, CO_2_ mainly interacts with the carbonyl group; however, after including the MMA and BA, CO_2_ is located close to both the OH group of HEMA and the carbonyl group of BA (see Supplementary Fig. S7a) and, thus, the largest increment of the binding energy (11.5 kJ·mol^−1^) is observed*.* The introduction of CO_2_ notably alters the molecular structure of the copolymer (see Supplementary Fig. S7b). Finally, in the copolymer including NaSS, the presence of MMA/BA allows the CO_2_ to locate in a position where π–π interactions with the benzene ring of functional monomer (i.e. NaSS) are favored, as in the copolymer with BS (see Supplementary Fig. S8).

#### Interaction of CO_2_ with GO/copolymer composites

In this section, the performance of the GO/copolymer composites in CO_2_ capture is analyzed for the four copolymers that showed the highest affinity with CO_2_, concretely, those which include AEMH, GMA, HEMA, and NaSS functional monomers. In this case, the interaction energy between the GO platelet and the copolymer was estimated, as in the previous section, by using the ZPVE-corrected energies (*E*_*0*_):

2 $$\Delta E_{{\text{int} }} = E_{0} ({\text{composite}}) - E_{0} ({\text{GO}}) - E_{0} ({\text{copolymer}})$$

The results were collected in Table 4. As in the case of the values from Table 3, calculated energies are similar to those measured and calculated for GO and its composite with polymers^15, 33, 34^, which indicates the reliability of the results. It is remarkable how the binding energy is increased for the composite including GMA (from − 18.3 to − 54.0 kJ·mol^−1^), while a softer increase is found for AEMH (from − 14.7 to − 19.3 kJ·mol^−1^). However, the binding energy is decreased for the composites including NaSS (from − 33.4 to − 23.1 kJ·mol^−1^) and HEMA (from − 24.2 to − 17.0 kJ mol^−1^). These last two cases are striking since it is well known that GO is a good CO_2_ adsorbent^11^ and, therefore, a cooperative effect is expected, enhancing the capacity of the copolymer.

**Table 4:** Binding energy of CO_2_ with GO/copolymer composite (Δ*E*_CO2_), in kJ·mol^−1^. Distance between CO_2_ and the composite (*D*), in Å. C=O bond length (*R*_i_), in Å, and O=C=O angle (*α*), in degrees, of CO_2_ molecule. Interaction energy between GO and the copolymer (Δ*E*_int_), in kJ·mol^−1^.

	AEMH	GMA	NaSS	HEMA
Δ*E*_CO2_	− 19.3	− 54.0	− 23.1	− 17.0
*D*	2.470	1.920	2.402	2.700
*R*_1_	1.171	1.176	1.165	1.165
*R*_2_	1.160	1.158	1.165	1.165
α	175.5	176.7	176.1	177.6
Δ*E*_int_	− 172.2	− 137.7	− 224.8	− 115.7

The composite with GMA presents the highest binding affinity for CO_2_ (− 54.0 kJ·mol^−1^). Without CO_2_, the copolymer is oriented in such a way that the carbonyl group of MMA is close to one of the carboxyl groups of GO. The interaction energy of GO and the copolymer is − 137.7 kJ·mol^−1^. However, the introduction of CO_2_ significantly changes the positioning of the copolymer and, thus, after the adsorption, the epoxy group of GMA is now able to interact with the acid group of GO, with an O_epox_–H_COOH_ distance of only 1.721 Å. The CO_2_ is placed between the carboxyl group of GO and the carbonyl group of MMA and, thus, a cooperative effect between GO and the copolymer is observed (see Supplementary Fig. S9).

When the AEMH-containing copolymer is placed in the GO platelet, the binding energy is increased to a lower extent than in the case of GMA. AEMH is placed in such a way that the Cl^‒^ ion interacts with the OH group of the GO surface, while the amino group is close to the carboxyl group. This makes the copolymer-GO interaction energy to be notably higher (− 172.2 kJ·mol^−1^) than for the GMA copolymer. The molecule of CO_2_ interacts mainly with the amino group of AEMH, as in the copolymer without GO, weakening the GO-copolymer interaction (the Cl^−^⋯HO distance increases from 2.259 to 2.284 Å). Thus, in the presence of CO_2_, the copolymer is attached to GO mainly by the interaction between the carbonyl group of AEMH and the nearby carboxyl group. The small increase observed in the binding energy can be due to a rather small model of GO. In Supplementary Fig. S10 can be observed how the copolymer is covering the whole GO platelet and, therefore, the CO_2_ is able to interact mainly only with the copolymer, in such a way that the effect of GO is almost absent. A larger model probably would increase this binding energy.

In the case of NaSS, there is a strong interaction between the copolymer and GO. Na^+^ is placed near the epoxy group and one of the oxygens from the sulfonate group interacts with the carboxyl group of GO. Also, BA moiety is oriented so that the carbonyl group is in the vicinity of another epoxy group of the GO surface. The distance between Na^+^ and the epoxy group is increased after CO_2_ adsorption, i.e. Na^+^ moves towards the carboxyl group. Besides, the sulfonate anion moves further from the COOH and C=O groups of GO. In the composite, CO_2_ is placed closer to the sulfonate group, while in the isolated copolymer there is an interaction with the carbonyl of BA and the aromatic ring of NaSS. Thus, the increase in the interaction energy provided by the GO platelet is compensated by a weaker interaction with the copolymer, and the net effect is a small decrease in binding energy (see Supplementary Fig. S11).

Finally, the HEMA composite presents the lowest binding affinity toward CO_2_. Also, of all four investigated composites, the one with HEMA has the lowest interaction energy between the GO platelet and the copolymer. The copolymer is placed in such a way that OH groups of HEMA and GO form hydrogen bonds, while oxygen atom from ether moiety of the carboxyl group of MMA forms another with the carboxyl group of GO. CO_2_ is positioned close to the hydroxyl group of HEMA and in relatively close vicinity of the carbonyl group of BA (see Supplementary Fig. S12). After the adsorption, the copolymer moves further from GO. For instance, two mentioned hydrogen bonds change the length from 2.050 to 2.124 Å and from 1.831 to 1.843 Å, respectively. As in the case of NaSS, CO_2_ is positioned far from the GO and interacts mainly with the copolymer. Therefore, as a result, binding energy decreases.

#### Interaction of N_2_ with GO/copolymer composites

In this subsection CO_2_/N_2,_ selectivity of investigated materials was estimated. More precisely, energies by which four composites selected in the previous subsection bind N_2_ were calculated. The results were collected in Table 5. The composite with AEMH binds N_2_ with the lowest energy. The nitrogen molecule is positioned close to the butyl group of BA and the carbonyl group of MMA (see Supplementary Fig. S13). Similarly, in the case of NaSS, N_2_ is placed between the butyl group of BA and one of the oxygens from the sulfonate group, to which it is close (see Supplementary Fig. S14). Therefore, the binding energy is only slightly higher than in the previously discussed composite. In the composite with HEMA, bonding occurs through the ether group of HEMA and carbonyl group of BA (see Supplementary Fig. S15), and thus, since N_2_ is placed close to the two heteroatoms (i.e. oxygens), the energy of adsorption is higher than in the case of the AEMH and the NaSS. Lastly, the composite with GMA has the highest binding energy, and nitrogen is placed close to the oxygens of the ether group of GMA and carbonyl group of BA (see Supplementary Fig. S16). For any of the composites, the introduction of N_2_ does not remarkably change the relative position of the copolymer in the composite. The position at which CO_2_ is primarily bound differs from that where N_2_ is adsorbed. Moreover, in the case of GMA and AEMH, these two molecules bind CO_2_ at significantly different positions.

**Table 5:** Binding energy of N_2_ with GO/copolymer composite (Δ*E*_N2_), in kJ·mol^−1^. Distance between N_2_ and the composite (*D*), in Å. N$\equiv$N bond length (*R*) of N_2_ molecule.

	AEMH	GMA	NaSS	HEMA
Δ*E*_N2_	− 6.8	− 9.3	− 7.0	− 8.6
*D*	2.737	2.809	3.003	2.741
*R*	1.101	1.101	1.101	1.101

## Experimental results

For the experimental work, GMA, AEMH, HEMA, and NaSS were selected as functional monomers added in a small amount (3%) to the main monomer mixture made of MMA/BA, to produce functionalized polymer nanoparticles. Table 6 presents the characteristics of the polymer particles. Despite that the syntheses were performed under identical conditions, the polymer dispersions have distinct features, which means that the type of functional monomer affected the polymerization process and the polymer properties. NaSS-, GMA- and HEMA-functionalized particles have a similar average particle size of about 80 nm, whereas AEMH functionalized particles are much larger and polydispersed. Obviously, the last system was less colloidally stable during emulsion polymerization, likely due to the cationic surfactant employed for colloidal stabilization in order to avoid possible ionic interactions between the surfactant and AEMH. As a consequence of the larger average size of AEMH functionalized particles, shorter polymer chains were produced due to the lower number of radicals per particle, reducing the possibility of bimolecular termination. The THF insoluble polymer fraction (gel fraction) that indicates the presence of cross-linked and branched structures was very low. The gel fraction of NaSS-containing polymer was slightly increased, which is probably the result of the incorporation of sulfonate moieties within MMA/BA chains, lowering their solubility in THF, especially high molar mass chains. GMA- and HEMA-containing polymers presented an increased amount of insoluble fraction, likely corresponding to crosslinked and branched structures.

**Table 6:** Characteristic of polymer particles.

	Molar mass M_w_ (Da)	Polydispersity	THF insoluble fraction (gel) (%)	Particle size (nm)
MMA/BA/NaSS	796,524	3.58	12	81.5 ± 0.2
MMA/BA/GMA	1,284,206	3.6	24	86.7 ± 1.2
MMA/BA/AEMH	428,966	4.31	< 5	161.6 ± 1.2
MMA/BA/HEMA	648,523	2.08	46	93.5 ± 0.61

**Figure 3:**
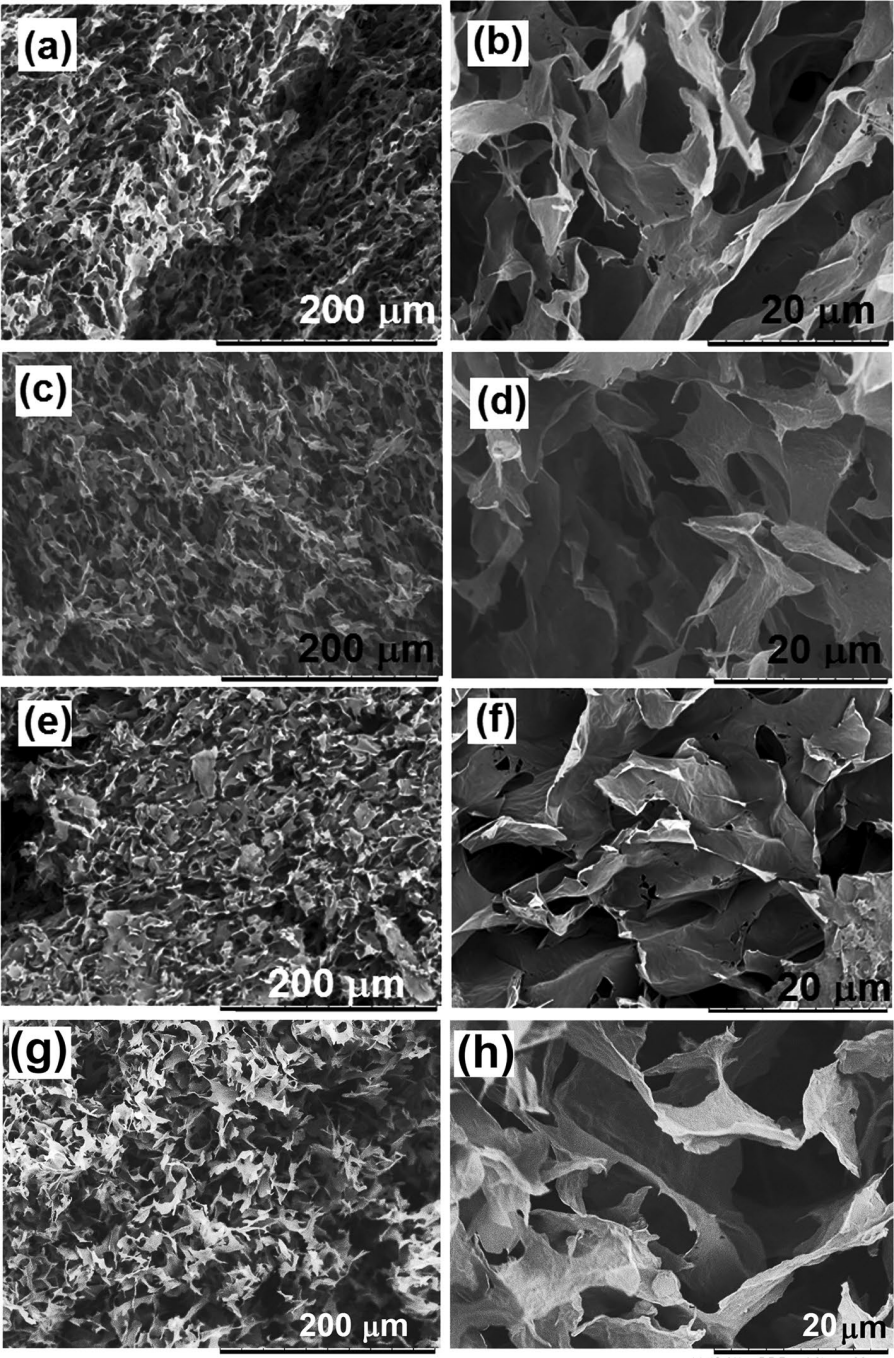
Morphology of the GO/polymer composites with different functional monomers under different magnification: (**a**) and (**b**) NaSS; (**c**) and (**d**) GMA; (**e**) and (**f**) AEMH; (**g**) and (**h**) HEMA.

The nanocomposite structures were created by mixing the functionalized polymer particles dispersion with GO nanoplatelets dispersion, during which process the polymer particles were adsorbed onto the platelet surface. By gravimetrical analysis of resultant water, after the composite platelets were recuperated, it was shown that the whole amount of polymer from the dispersion was incorporated within the composite structure. Therefore, the resulting composites contain GO and polymer in a 1:0.5 weight ratio. In Fig. 3, the morphology of the nanocomposites determined by SEM is presented under two magnifications. Porous materials were obtained in all cases, probably due to the hydrophobic interaction of the composite rGO-polymer platelets placed in aqueous dispersion. Namely polymer nanoparticles interacted with the oxygen functional groups at the GO surface, creating H bondings (H donor groups such as COOH and OH in GO interacted with H accepting groups in polymers, such as carbonyl, sulfonate, and amino). As the oxygen functional groups on GO provide amphiphilic character to GO, by decreasing the number of these functionalities, GO platelets became more hydrophobic. This induced aggregation, wrinkling, and crumpling of the platelets, forming the porous structure, which processes were even promoted during drying. However, the presence of different functional monomers, even in as low amounts as 3 wt% with respect to polymer, influences the structure and morphology. Placed onto the polymer particles surface, the different functionalities affected polymer—GO interactions. While nanocomposites functionalized with NaSS, GMA, and HEMA (Fig. 3a,c,g, respectively) have fluffy structures with very well-developed pores of about 5–10 μm, the nanocomposite functionalized with AEHM is less porous (Fig. 3e). The cationic nature of the last probably induced ionic complexing with the numerous anionic oxygen-containing functional groups of GO, giving rise to more compact composite structures. Under higher magnification (Fig. 3b,d,f,h), no important differences between the four nanocomposites may be noticed, which creates a stable platform for comparison of the interaction forces between CO_2_ and the respective functionalities in the nanocomposites.

**Figure 4:**
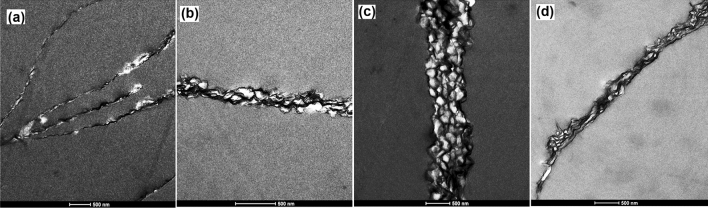
TEM images of (**a**) NaSS; (**b**) GMA; (**c**) AEMH; and (**d**) HEMA functionalized composites. Scale bars in all images are 500 nm.

The structure of the nanocomposites was evaluated by TEM imaging (Fig. 4), where the cross-section of the composite platelets may be observed. The black areas represent the GO platelets, whereas the white areas correspond to the polymer. A peculiar combination of these two phases may be observed, in which the platelets wrap the single polymer particle or aggregates of a few of them, creating composite honeycomb-like structures. The presence of GO platelets probably prevented the full particle coalescence and formation of large polymer areas. The thickness of the composite platelets depends on the functional monomer, therefore, the composite platelets functionalized with NaSS present a thickness of about 200 nm, those functionalized with GMA and HEMA have a thickness of 250–300 nm, whereas the AEMH functionalized platelets, with a thickness of 500–1000 nm, are the thickest. Two possible causes can affect the formation of thicker AEMH-containing composite platelets. On one hand, the size of AEMH functionalized polymer particles is double on average in comparison to other functionalized particles, and on the other, the ionic interactions between cationic polymer particles and anionic GO increased the likelihood of platelets aggregation.

TGA curves of the nanocomposites, shown in Supplementary Fig. S17, were used to determine the content of oxygen functionalities in each composite. Thermal degradation occurred in three steps, assigned as follows. The humidity is lost until 100 °C, the weight loss between 100 and 260 °C corresponds to a loss of oxygen functionalities distributed onto GO, whereas the polymer is degraded between 300 and 400 °C^23^. The advantage that the oxygen functionalities over GO are lost in the distinct region than the polymer itself (including the functionalities containing oxygen within the polymer chains), provided the possibility to calculate their relative contents. The content of oxygen functionalities (originating from GO) within the composites and their textural properties are presented In Table 7. The quantity of the oxygen functional groups is similar in all composites.

**Table 7:** Textural properties of the composite platelets.

Material	% O-functionality	BET Surface area (cm^2^ g^−1^)	Total pore volume (cm^3^ g^−1^)	Micropore volume (cm^3^ g^−1^)	Average pore width (nm)
GO/pol-NaSS	15.4	22	0.0364	0.0027	13
GO/pol-GMA	13.3	48	0.0878	< 0.001	12
GO/pol-AEMH	12.5	50	0.081	< 0.001	10
GO/pol-HEMA	14.7	77	0.1249	< 0.001	12

Relatively modest specific BET surface area was observed for all nanocomposites (Table 7), which is not surprising, as the synthesis of the nanocomposites was altered in order to limit the development of the porous structures and to provide a base to investigate the effect of functionalities on the CO_2_ selective capture. The observed porosity in SEM images is surface morphology on the micron level, while the textural properties from Table 7 demonstrate that no deep meso- and micropores were developed. NaSS functionalized composite presents the lowest BET surface area and total pore volume, indicating that this composite is less porous and more compact than others, which might be due to the aromatic ring from NaSS functional monomer that interacts more tightly with GO than other polymers. Despite this, only NaSS functionalized composite is characterized by microporosity, although in small quantity. It might provide compensation in the case of CO_2_ adsorption, as it is known that microporosity is one of determining characteristics of the CO_2_ adsorption capacity^23^. The textural properties of GMA and AEMH composites are similar.

**Figure 5:**
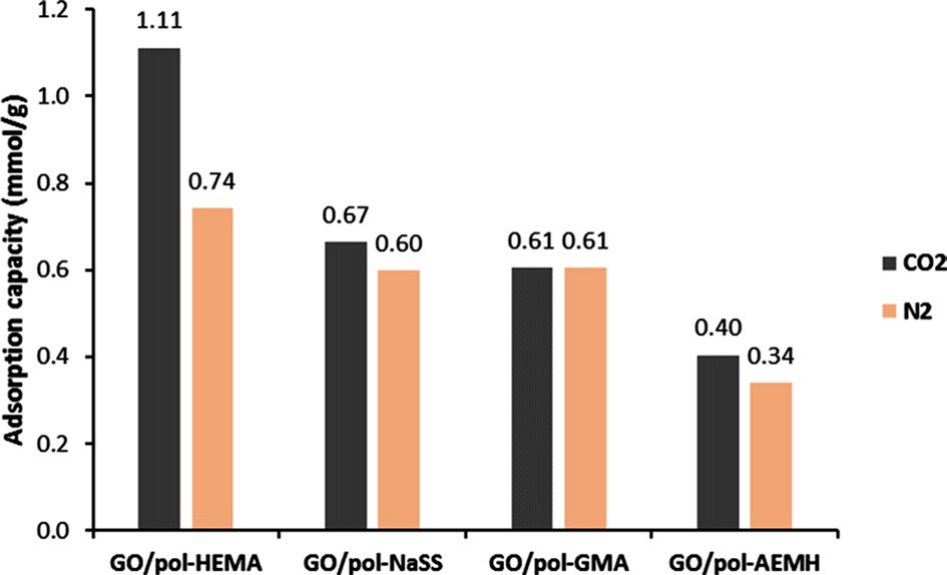
Adsorption of CO_2_ and N_2_ of the functionalized GO/polymer composites.

The CO_2_ and N_2_ adsorption capacity of nanocomposites is given in Supplementary Table S1 and in Fig. 5. For comparison, the CO_2_ adsorption of neat polymers is also shown. Taking into account that the gas adsorption was studied at atmospheric conditions, CO_2_ adsorption is within the range typical for carbon-based nanomaterials (0.5–1 mmol g^−1^), but it is still much lower than those we previously achieved by similar materials (3.7 mmol g^−1^)^24^. Surprisingly, contrary to the previous study^24^, N_2_ adsorption was very similar to that of CO_2_ adsorption, indicating that nanocomposites have very low selectivity. The modest capacity for selective CO_2_ capture of the present nanocomposites might be due to a few reasons. The most important for this study is that the composite synthesis was directed to obtain possible similar materials and thus to provide a good basis for the comparison using theoretical studies.

The amount of CO_2_ adsorbed by neat polymer materials is approximately one order of magnitude lower than that adsorbed by nanocomposites. (Supplementary Table S1). Considering that the polymers are not porous, this result is not surprising. Nevertheless, it is clear that a combination of polymers with GO might be a useful way to increase their CO_2_ adsorption performance.

The comparison of the experimental results with the theoretical prediction of the binding energy between nanocomposites and CO_2_ is not straightforward. Even with the intention to eliminate the effect of the textural characteristics of the synthesized nanocomposites, only GMA and AEMH functionalized nanocomposites presented similar porous morphology, providing a basis for comparison. According to Table 4, GMA functionalized composite presents the highest binding energy, much higher than that of AEMH (54 kJ mol^−1^ versus 19 kJ mol^−1^). According to Supplementary Table S1, GMA composite adsorbs 0.7 mmol g^−1^, whereas AEMH composite adsorbs 0.5 mmol g^−1^, thus, the same trend is followed. On the other hand, Table 5 presents that GMA-based composite has N_2_ binding energy of almost 10 kJ·mol^−1^, whereas AEMH composite only 7 kJ·mol^−1^. The same trend is followed by experimental results, thus GMA composites adsorbed 0.61 mmol g^−1^ of N_2_, and AEMH adsorbed 0.34 mmol·g^−1^. Therefore if, by appropriate design of synthesis procedure, the effect of morphology over the gas absorption capacity of the material can be eliminated, a comparison with the theoretical prediction of the adsorption can be performed. In such a case, the presented theoretical study seems to be an excellent tool to predict the interaction of functionalized composite structures with CO_2_ and N_2_, which can be useful for the selection of functionalization of composites for application in gas adsorption.

Moreover, the comparison of the theoretical prediction and experimental results for HEMA composites provides evidence of the importance of the porous structures’ morphology for the selective CO2 capture. According to Table 4, HEMA functionalized composite presented three-time lower binding energy for CO_2_ and slightly lower N_2_ binding energy, as shown in Table 5. Experimental results show that this composite presents the highest CO_2_ adsorption (1 mmol·g^−1^) and the highest selectivity over N_2_ (Fig. 5). Considering the textural properties of HEMA composites presented in Table 7, i.e. the highest BET surface area and total pore volume, this composite is clearly the most porous when compared to the other studied nanocomposites in this work, and accordingly, it achieved the highest performance for selective CO_2_ capture.

## Conclusions

We have performed a computational and experimental study in the pursuit of new GO/copolymer composites that may be promising candidates for CO_2_ capture materials. In particular, the interactions between CO_2_ and seven functional monomers, namely, acrylamide (Am), 2-aminoethyl methacrylate hydrochloride (AEMH), methyl 2-chloro acrylate (MClA), hydroxyethyl methacrylate (HEMA), glycidyl methacrylate (GMA), sodium 4-vinylbenzenesulfonate (NaSS), and 4-bromostyrene (BS), have been studied by means of density functional theory (DFT) calculations. The binding energies with CO_2_ were analyzed first for the isolated functional monomers and then for the copolymers with MMA/BA, in terms of the interactions with the different moieties and structural rearrangements. The incorporation of MMA/BA enhances the CO_2_ affinity due to the presence of the carbonyl groups of the MMA/BA copolymer, allowing for new interactions with CO_2_. The only exception is Am, in which case CO_2_ does not interact with carbonyl groups. From this study, four copolymers including AEMH, GMA, HEMA, and NaSS have been selected for analysis of adsorption of the GO/polymer composites.

The calculations show that the binding energy for CO_2_ is increased for the composites including AEMH and GMA, especially for the latter, while for NaSS and HEMA is decreased. This striking result, where the cooperative effect of the GO seems to be absent, may be explained by a remarkably strong interaction between the copolymer and GO, in such a way that the CO_2_, while having limited interaction with GO, now shows a less effective interaction with the copolymer.

These four composites have also been synthesized experimentally and the CO_2_ selective adsorption over N_2_ has been evaluated. Even with a synthesis procedure designed to decrease the development of highly porous structures, only GMA and AEHM functionalized composites presented very similar textural structures allowing us to study the effect of different functionalities on the selective CO_2_ capture. The trends observed experimentally were exactly predicted by the theoretical study. Moreover, the difference between the theoretical and experimental results in the case of HEMA functionalized composite confirmed the importance of the effect of morphology. In summary, this work shows that DFT is a useful tool for screening functional monomers for the design and synthesis of new GO-based materials for CO_2_ capture.

## Supplementary Information

Supplementary Information.

## Data Availability

Some of the datasets used and/or analyzed during the current study are presented in the Supplementary Information file. The datasets used and/or analyzed during the current study, which are not shown in the Supplementary Information file, are available from the corresponding author upon reasonable request.
