# Supplementary material for: Experimental and theoretical study of the effect of different functionalities of graphene oxide/polymer composites on selective CO2 capture
Source: Sci Rep. 2022 Sep 26;12:15992. doi: 10.1038/s41598-022-20189-5 (PMC9512785; doi:10.1038/s41598-022-20189-5)
Supplement: Supplementary file 1 — Supplementary Information. [file 41598_2022_20189_MOESM1_ESM.pdf]

Experimental and theoretical study of the effect of different functionalities of graphene oxide/polymer composites on selective CO2 capture

Branislav Stankovic[a,b], Iranzu Barbarin[a], Oihane Sanz,[a] Radmila Tomovska[a,c,*] and Fernando Ruipérez[d,*]

[a]*POLYMAT and Departamento de Química Aplicada, Facultad de Ciencias Químicas, University of the Basque Country UPV/EHU, Joxe Mari Korta Zentroa, Tolosa Hiribidea, 72, 20018 Donostia-San Sebastián, Spain;*

[b]*Faculty of Physical Chemistry, University of Belgrade, Studentski Trg 12-16, Belgrade, 11050 Republic of Serbia*

[c]*IKERBASQUE, Basque Foundation for Science, María Díaz de Haro 3, 48013 Bilbao, Spain*

[d]*POLYMAT and Physical Chemistry Department, Faculty of Pharmacy, University of the Basque Country, 01006 Vitoria-Gasteiz, Spain*

Content:

1. **Figure S1** Optimized geometry of the GO.
2. **Figure S2** Optimized geometry of the MMA/BA/Am + CO_2 complex.
3. **Figure S3** Optimized geometry of the MMA/BA/AEMH + CO_2 complex.
4. **Figure S4** Optimized geometry of the MMA/BA/BS + CO_2 complex.
5. **Figure S5** Optimized geometry of the MMA/BA/MClA + CO_2 complex.
6. **Figure S6** Optimized geometry of the MMA/BA/GMA + CO_2 complex.
7. **Figure S7** (a) Optimized geometry of the MMA/BA/HEMA + CO_2 complex. (b) geometry of the copolymer without CO_2.
8. **Figure S8** Optimized geometry of the MMA/BA/NaSS + CO_2 complex.
9. **Figure S9** Optimized geometry of the GO/MMA/BA/GMA + CO_2 complex.
10. **Figure S10** Optimized geometry of the GO/MMA/BA/AEMH + CO_2 complex.
11. **Figure S11** Optimized geometry of the GO/MMA/BA/NaSS + CO_2 complex.
12. **Figure S12** Optimized geometry of the GO/MMA/BA/HEMA + CO_2 complex.
13. **Figure S13** Optimized geometry of the GO/MMA/BA/AEMH + N_2 complex.
14. **Figure S14** Optimized geometry of the GO/MMA/BA/NaSS + N_2 complex.
15. **Figure S15** Optimized geometry of the GO/MMA/BA/HEMA + N_2 complex.
16. **Figure S16** Optimized geometry of the GO/MMA/BA/GMA + N_2 complex.
17. **Figure S17** TGA curves of the functionalized GO-polymer composites.
18. **Table S1** CO_2 and N_2 adsorption by the neat functionalized polymers and functionalized GO-polymer composites obtained at T = 25 ^oC and P = 1 atm.

1. Figures:

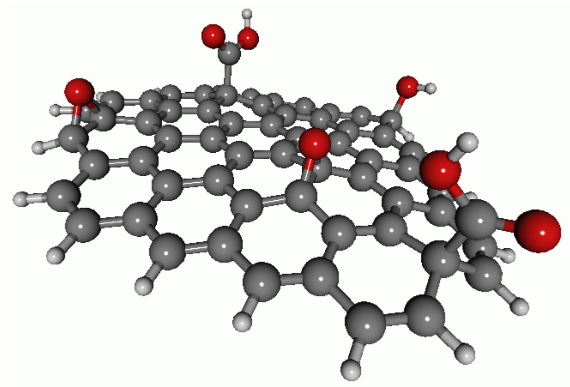

Figure S1 Optimized geometry of the GO.

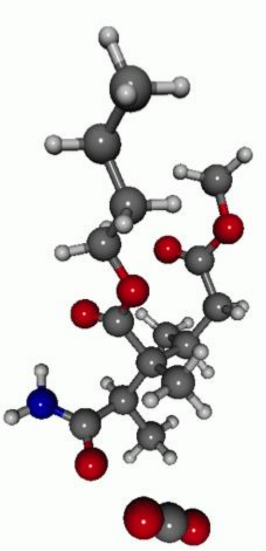

Figure S2 Optimized geometry of the MMA/BA/Am + CO_2 complex.

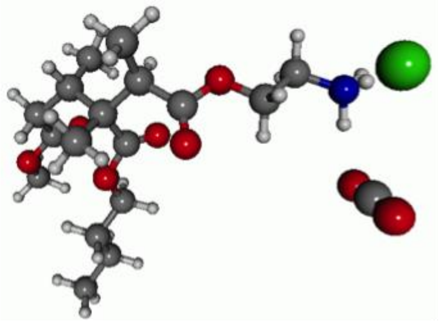

Figure S3 Optimized geometry of the MMA/BA/AEMH + CO_2 complex.

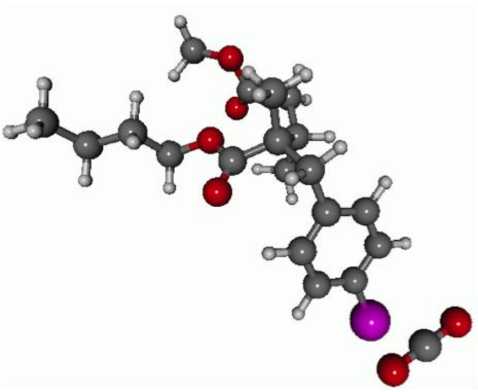

Figure S4 Optimized geometry of the MMA/BA/BS + CO_2 complex.

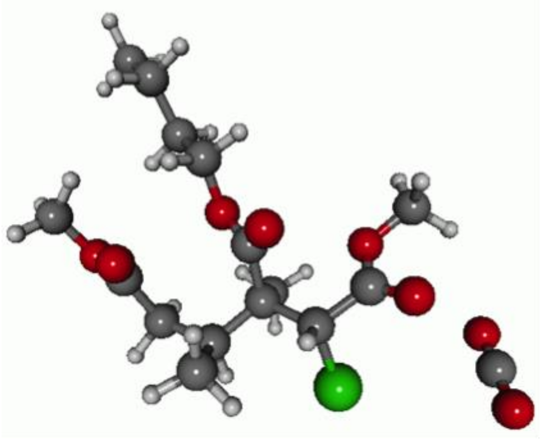

Figure S5 Optimized geometry of the MMA/BA/MClA + CO_2 complex.

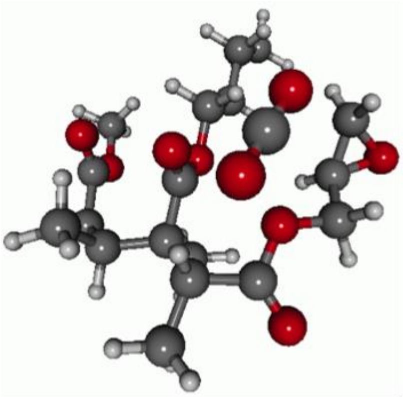

Figure S6 Optimized geometry of the MMA/BA/GMA + CO_2 complex.

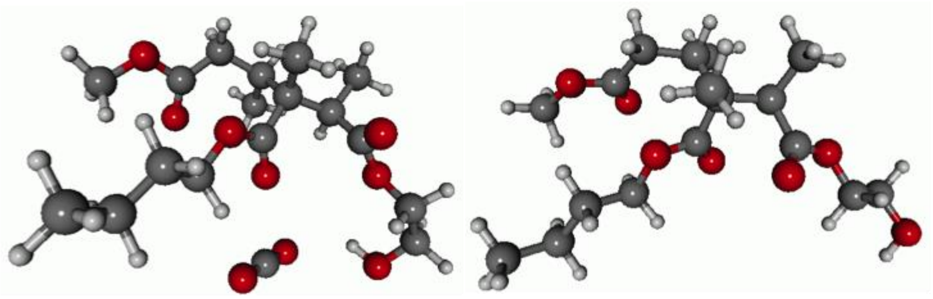

Figure S7 (a) Optimized geometry of the MMA/BA/HEMA + CO_2 complex. (b) geometry of the copolymer without CO_2.

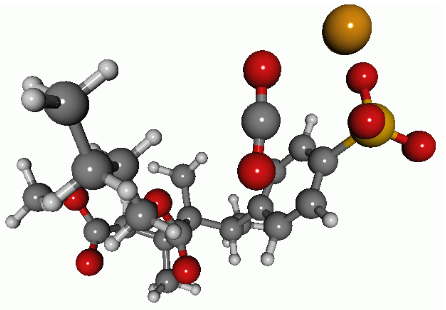

Figure S8 Optimized geometry of the MMA/BA/NaSS + CO_2 complex.

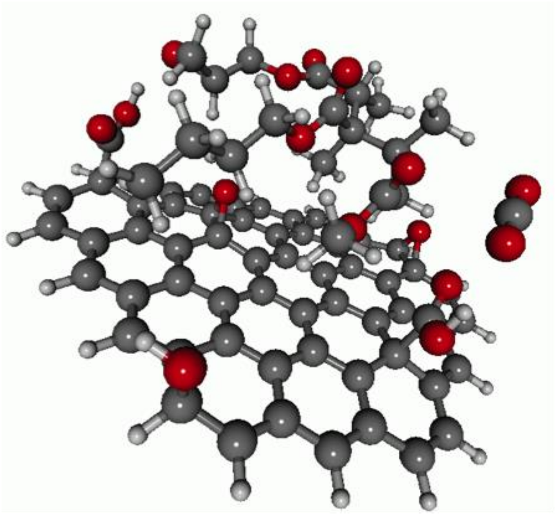

Figure S9 Optimized geometry of the GO/ MMA/BA/GMA + CO_2 complex.

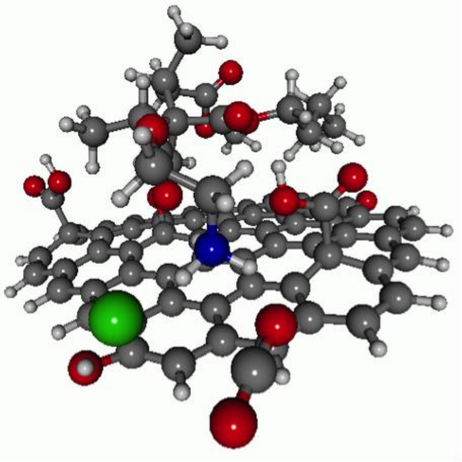

Figure S10 Optimized geometry of the GO/ MMA/BA/AEMH + CO_2 complex.

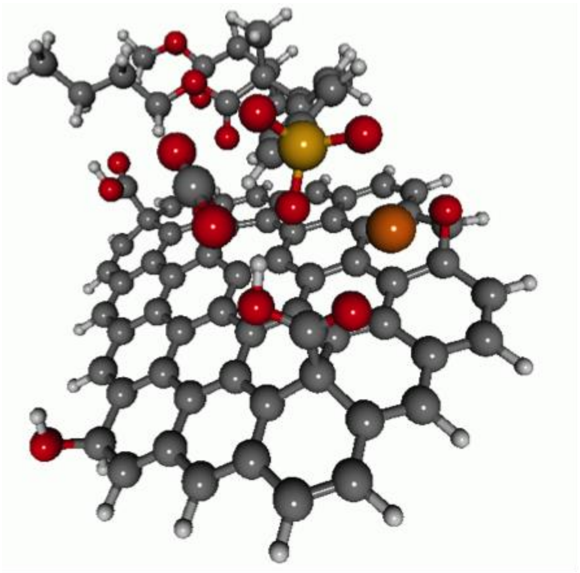

Figure S11 Optimized geometry of the GO/ MMA/BA/NaSS + CO_2 complex.

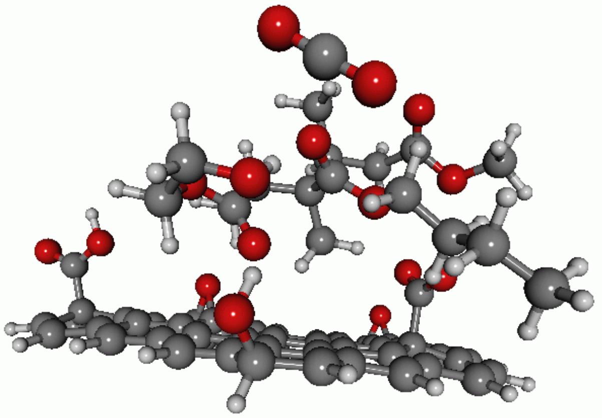

Figure S12 Optimized geometry of the GO/MMA/BA/HEMA + CO_2 complex.

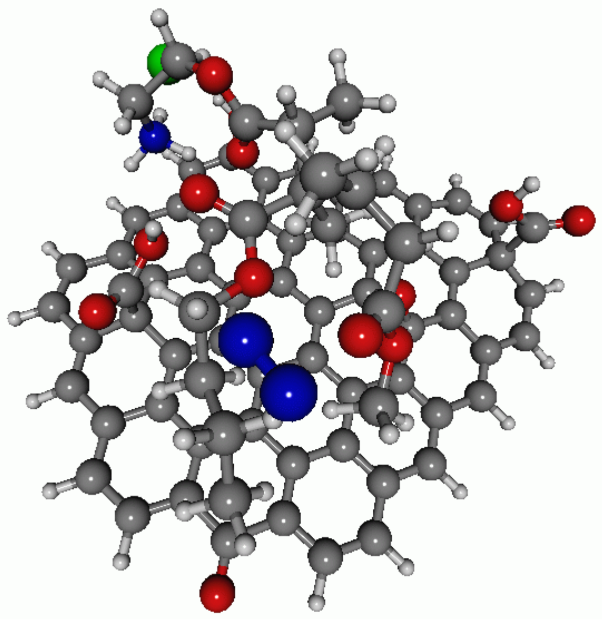

Figure S13 Optimized geometry of the GO/MMA/BA/AEMH + N_2 complex.

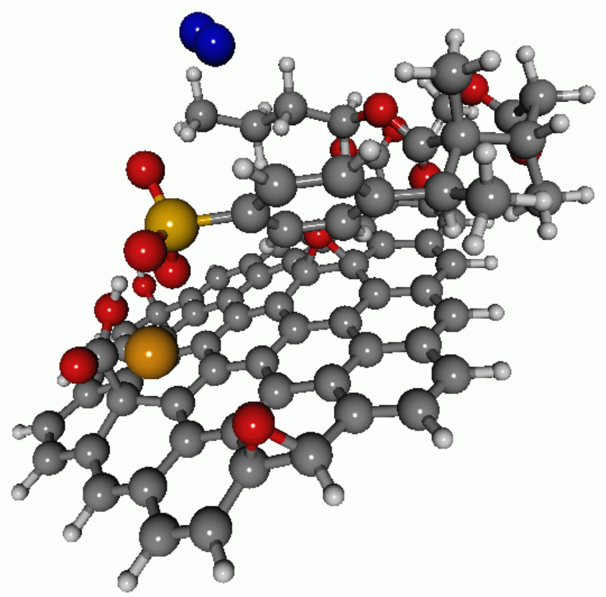

Figure S14 Optimized geometry of the GO/MMA/BA/NaSS + N_2 complex.

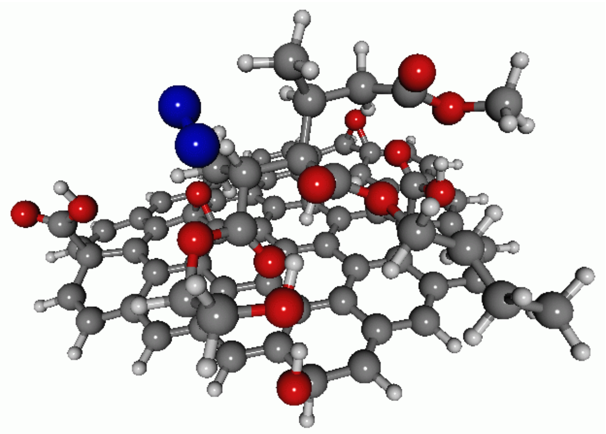

Figure S15 Optimized geometry of the GO/MMA/BA/HEMA + N_2 complex.

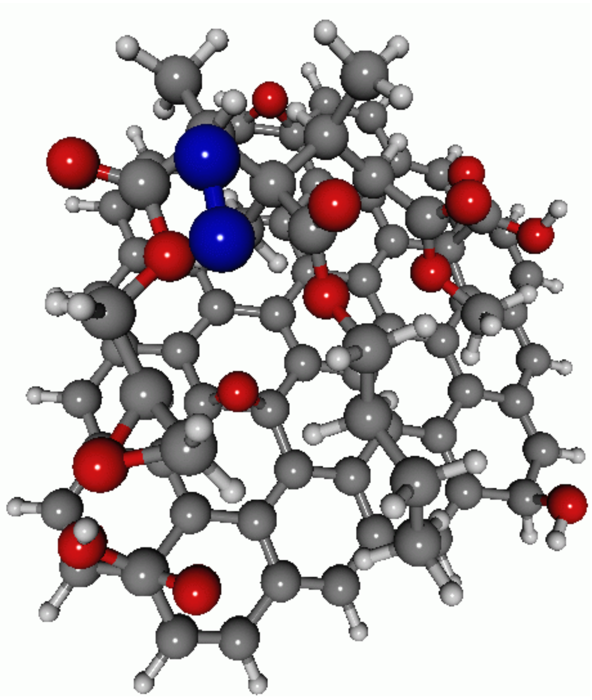

Figure S16 Optimized geometry of the GO/MMA/BA/GMA + N_2 complex.

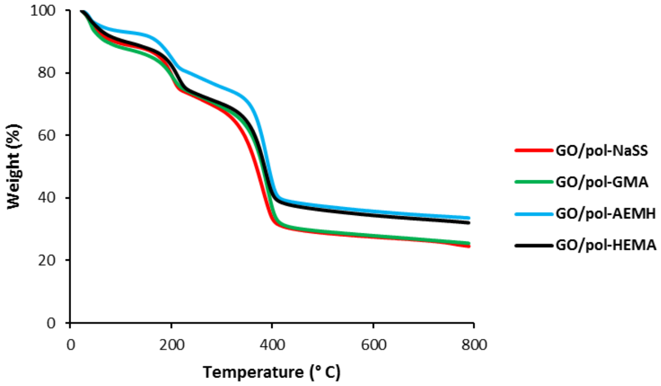

Figure S17 TGA curves of the functionalized GO-polymer composites.

2. Tables:

Table S1 CO_2 and N_2 adsorption by the neat functionalized polymers and functionalized GO-polymer composites obtained at T=25°C and P=1 atm.

Composite	CO_2 adsorption (mmol/g)		N_2 adsorption (mmol/g)
	Neat polymer	GO/polymer	GO/polymer
**GO/pol-NaSS**	0.067	0.67	0.60
**GO/pol-GMA**	0.057	0.61	0.61
**GO/pol-AEMH**	0.062	0.40	0.34
**GO/pol-HEMA**	0.076	1.11	0.74
